# Individual differences in scientists’ aesthetic disposition, aesthetic experiences, and aesthetic sensitivity in scientific work

**DOI:** 10.3389/fpsyg.2023.1197870

**Published:** 2024-01-08

**Authors:** Christopher Jacobi, Peter J. Varga, Zohaib Jessani, Brandon Vaidyanathan

**Affiliations:** ^1^Department of Sociology, The Catholic University of America, Washington, DC, United States; ^2^Department of Experimental Psychology, University of Oxford, Oxford, United Kingdom; ^3^Department of Psychology, The Catholic University of America, Washington, DC, United States

**Keywords:** aesthetics, Big Five, scientists, beauty, aesthetic sensitivity, wonder, awe, dispositional aesthetics

## Abstract

**Introduction:**

The role of personality in shaping engagement with aesthetics in science has been almost entirely unexplored. Whereas artists and arts settings (e.g., museums) are well-studied from a psychological perspective, the practice of science has often been seen as purely rational or dry. In response, this study presents novel findings on the critical role of scientists’ individual differences, which shape their engagement with aesthetics, such as the frequency of their experiences of beauty, wonder, and awe in their scientific work.

**Methods:**

Based on a very large and representative four-country study of scientists in the fields of biology and physics (*N* = 3,092), this study analyzed the associations of Big Five personality traits among scientists with (i) dispositional aesthetics (DPES-awe), (ii) the frequency of aesthetic experiences in scientific work, and (iii) aesthetic sensitivity in science. These survey-weighted OLS regression models included extensive statistical controls for sociodemographic factors.

**Results:**

As hypothesized, openness is positively, and neuroticism is negatively linked with dispositional aesthetics, the frequency of aesthetic experiences in scientific work, and aesthetic sensitivity in science. Unexpectedly, agreeableness and conscientiousness (but not extraversion) are highly significant and strong predictors of the three trait and state aesthetic engagement variables.

**Discussion:**

The aesthetic engagement and personality framework of this paper is empirically supported and demonstrates the importance of personality types of scientists in the practice of science. The unexpectedly strong association of agreeableness with aesthetic engagement points to the importance of cooperation, collaboration, and communication to maximize scientific creativity.

## 1 Introduction

Individual differences have long been associated with variation in the type and frequency of people’s aesthetic engagement, but most of this work has been done in arts domains and arts-adjacent populations. In this article, we synthesize this literature to form an aesthetic engagement and personality framework that extends philosophical and theoretical approaches to elucidate the interaction between individuals’ Big Five personality traits and their experiences of beauty, wonder, and awe in broader, non-arts-specific context. Grounded in empirical evidence from research in psychology and empirical aesthetics, we posit a framework in which individual differences in aesthetic engagement—whether appreciating art, finding beauty in scientific theories, or engaging creatively with the environment—can largely be attributed to key personality traits, particularly those defined within the Big Five personality taxonomy.

Of these traits, openness to experience is given primary emphasis due to its consistent association with aesthetic engagement. Individuals with high levels of openness may be more disposed to perceive and engage with aesthetic aspects of their environment, which could in turn influence their experiences in a variety of contexts such as work, education, art, and even daily life activities. Inasmuch as aesthetic experiences are associated with flourishing (Jacobi et al., 2022), understanding the relationship and interactions between aesthetics and personality traits may be important for improving wellbeing outcomes. To begin exploring these directions, we apply our framework predicting the frequency of aesthetic experiences by Big Five personality traits to understanding the role of aesthetics in scientific engagement and creativity, particularly whether scientists with high trait openness might be more capable of appreciating aesthetic qualities in their work, possibly leading to heightened creativity, motivation, and retention in the field.

## 2 Literature and theory

The empirical study of individual differences and aesthetics is more than a century old, dating at least as far back as Fechner’s (1876) *Vorschule der Ästhetik* [Preschool of Aesthetics]. More modern research has examined aesthetics and personality in the contexts of taste and complexity (Eysenck, 1940), judgments of and preferences for visual art (Barron, 1953; Knapp and Wulff, 1963; Child, 1965), and even socio-political leanings (Wilson et al., 1973). Much of the psychological literature on this topic has focused on how specific personality traits predict certain kinds of aesthetic engagement (Rawlings et al., 2000; Keltner and Haidt, 2003; Thrash and Elliot, 2003; Shiota et al., 2007; Silvia et al., 2015; Atari et al., 2020) and how some personality traits correlate with aesthetics-adjacent domains, especially visual art (Locher, 2012; Afhami and Mohammadi-Zarghan, 2018; Pietras and Czernecka, 2018; Atari et al., 2020). Experiences of beauty, wonder, and awe have also been linked to improved wellbeing and quality of life (Wanzer et al., 2020; Jacobi et al., 2022) as well as with domains of self-transcendence, such as motivation and emotion (Marković, 2012). This research output shows a recent, growing interest in understanding the underlying personality traits that predict dispositional and state aesthetic emotion experiences (Shiota et al., 2006).

One of the most fruitful personality taxonomies for empirical aesthetics has been the Big Five, which includes openness to experience (preference for intellectual variety and novelty, including interest in aesthetics and art-related activities), agreeableness (tendency toward sympathy and helping others), extraversion (tendency toward sociability), conscientiousness (preference for organization and discipline), and neuroticism (lack of emotional stability and control) as its core dimensions (Costa and McCrae, 1992). Various studies have found associations between Big Five traits such as openness and conscientiousness and how individuals view the world, especially pertaining to aesthetic judgment and art interest (Afhami and Mohammadi-Zarghan, 2018). Among the Big Five traits, openness to experience emerges from both the theoretical and empirical literature as the most important to aesthetic engagement, encompassing aesthetic fluency and preferences (Chamorro-Premuzic, 2016) to appreciation of aesthetic quality (McManus and Furnham, 2006) and dispositional proneness to aesthetic emotions (Silvia et al., 2015; Fayn et al., 2018). Studies of the Big Five traits in the context of their association with aesthetics (Silvia et al., 2009; Fayn et al., 2015) show that openness has the strongest association with experiences of wonder, awe, and beauty and that the aesthetics facet of openness is perhaps its strongest indicator (Rawlings et al., 2000; McCrae, 2007; Atari et al., 2020). These associations might be partly attributable to the parallel association of openness with traits such as creativity, imagination, and curiosity (Silvia et al., 2009).

As a result of the consistency of these findings, openness is accepted as a central component of individual differences in aesthetic engagement, but the relations of the other four factors with aesthetics correlates is less clear. While openness is often correlated with creativity, imagination, and curiosity, it is also correlated with sensation-seeking (along with extraversion), which is another possible link between aesthetic engagement and personality-based predisposition to aesthetic engagement, such as awe (Keltner and Haidt, 2003; Chamorro-Premuzic et al., 2010; Fayn et al., 2018; Swami and Furnham, 2022). Openness was found to significantly predict preference for emotionally positive stimuli whereas neuroticism was found to predict preference for emotionally negative stimuli in a study of personality, aesthetic judgment of visual art, and emotional valence (Chamorro-Premuzic et al., 2010), suggesting that personality plays an important role in affective preference and predisposition.

Openness also relates to both aesthetic sensitivity and aesthetic fluency. Aesthetic sensitivity is the capacity to recognize aesthetic qualities in various contexts (Myszkowski, 2022). Traditionally conceptually adjacent to Eysenck’s (1940, 1992) “taste” factor, aesthetic sensitivity has been shown to vary between individuals and covary with other factors affecting aesthetic preference (Child, 1962). It is often correlated with aesthetic fluency, which is knowledge-based familiarity with the terms used to describe aesthetic experiences, because it provides more and varied contexts in which potential aesthetic stimuli can be conceptually and linguistically identified and labeled as such (Atari et al., 2020). These context-specific terms could conceivably vary by discipline such that scientists might uniquely appreciate the beauty of discovery as an aesthetic experience (e.g., Ritz and Vaidyanathan, 2023), but the sensitivity to that experience might vary on an individual level in tandem with personality.

In that vein, openness has been found to be the only Big Five trait that consistently predicts aesthetic fluency (Silvia, 2007; Chamorro-Premuzic et al., 2009, 2016; Atari et al., 2020). Openness has been shown to be an important predictor of aesthetic fluency among artists in the US and other countries (Atari et al., 2020) as well as American college students (Silvia, 2007). In addition to the robust effects of openness, some studies have also found that low neuroticism (i.e., emotional stability) also predicted aesthetic fluency (Atari et al., 2020) while others reported no significant relationship between the two variables, instead finding only small additional effects for extraversion and conscientiousness (Silvia, 2007). Psychologists have also studied the association of the other Big Five traits and aesthetic engagement more broadly. Like examinations of aesthetic fluency, these studies show smaller, mixed associations between aesthetics and agreeableness, conscientiousness, extraversion, and neuroticism. Some studies have found that agreeableness and conscientiousness are negatively correlated with aesthetic experiences (Furnham and Chamorro-Premuzic, 2004), while others have found small, positive correlations (Silvia et al., 2015). Similarly, research on the association between aesthetic engagement and the Big Five traits of extraversion and neuroticism also found mixed results (Afhami and Mohammadi-Zarghan, 2018). Extraversion has been shown to predict appreciation of aesthetic quality (McManus and Furnham, 2006) while both positively and negatively predicting aesthetic judgments in different studies by the same researchers (Chamorro-Premuzic and Furnham, 2004, 2009; Furnham and Chamorro-Premuzic, 2004; Swami and Furnham, 2022). Neuroticism influences aesthetic judgment (Furnham and Walker, 2001) but also helps explain the association of aesthetic judgment and anxiety (Child, 1962). Other studies have employed similar questionnaire-based designs to examine associations between various Big Five factors and aesthetic preferences, but have found only weak and contradictory effects (see Swami and Furnham, 2014 for a review). All of these findings tend to vary by measure and type of sample studied, suggesting that it is important to consider the population and experiential context (i.e., modality of experience), along with all five of these personality traits, when understanding its link to aesthetic engagement.

While the literature on personality and aesthetic engagement has largely focused on adult general and arts-oriented samples that are associated with increased aesthetic activity (McManus and Furnham, 2006), there has been a dearth of research on what role these constructs play for individuals in non-arts areas who might utilize aesthetics to guide their work (Girod, 2007), such as scientists whose education and work is typically negatively associated with aesthetic activity (McManus and Furnham, 2006). Scientists are thus an ideal population at which to look closer in this context, because while their work relies on predominately objective techniques, they also may employ creativity, imagination, and curiosity as well as subjective constructs such as wonder and beauty (e.g., symmetry, elegance, simplicity, etc.) to inform and motivate their work (Girod, 2007; Vaidyanathan and Varga, 2020; MacArthur, 2021).

Aesthetic engagement is thus proposed to play a surprisingly important role in the lives of scientists. Research has suggested that aesthetic engagement fosters education and improves scientific communication (Girod et al., 2010), and theorists have also proposed that experiences of beauty and awe do indeed drive scientific research and shape theories (MacArthur, 2021). Building on that theory, recent empirical research has shown that scientists’ aesthetic experiences are associated with retention in the field and flourishing (Jacobi et al., 2022). It is therefore important to study the link between personality and aesthetic engagement among scientists as it may help explain whether some scientists are more aesthetically prone in general or more inclined to encounter beauty, wonder, and awe at work.

### 2.1 Present study

While the philosophical and affect literatures are well-developed in the area of aesthetic engagement and individual differences, the present paper aims to lay the foundation for a more robust personality psychological framework by grounding enquiry into non-artists’ experiences of beauty, wonder, and awe in the Big Five personality taxonomy. To accomplish this aim, we tested personality as a predictor of dispositional and state aesthetic engagement among a large group of international scientists using questionnaire-based measures of personality and aesthetics correlates consistent with precedent. Based on the prior research related to the Big Five trait of openness to new experiences and aesthetic fluency, the following hypotheses were proposed:

1. *Higher openness among scientists would be associated with higher levels of dispositional positive affect (as indicated by higher scores on the DPES-awe sub-scale)*. Even though the literature on neuroticism was mixed, on balance, it still seems likely that this personality trait would also be associated with lower levels of dispositional positive affect.

While the first hypothesis referred to scientists’ trait aesthetic disposition, the second and third hypotheses refer to state aesthetic experiences of scientists in their scientific work.

2. *Higher openness among scientists would be associated with a higher prevalence of aesthetic experiences in scientific work (as indicated by higher scores on a frequency of aesthetic experiences in scientific work scale, which ranges from “never” to “weekly or more” frequent ones, while averaging across domains of beauty, wonder, and awe)*.
3. *Higher openness among scientists would be associated with more aesthetic sensitivity (as indicated by more identified contexts in which “beauty, wonder, and, awe” are experienced in science)*. Higher neuroticism would be associated with less aesthetic sensitivity in science.

The associations of the other three personality traits (agreeableness, conscientiousness, extraversion) with aesthetics in science were not hypothesized—because the literature was not as clear—but were investigated exploratively. While existing literature on aesthetics in science has highlighted the importance of disciplinary differences (e.g., MacArthur, 2021), it has largely overlooked the possible role of individual differences in shaping aesthetic predispositions. The literature on the relationship between personality and aesthetics, while unexamined in specific domains such as science, leads us to expect that associations found in previous studies will likely be found within the scientific community as well. If such differences are found, then they could have implications for understanding motivation, retention, mentoring relationships, and other factors affecting scientists’ ability to thrive in their work environments.

## 3 Methods

### 3.1 Participants and procedure

This data is part of a large empirical study of biologists and physicists at doctorate-granting institutions and research institutes in the United States, United Kingdom, Italy, and India who completed online questionnaires (analytic *N* = 3,092; starting *N* = 3,442). Physicists and biologists were chosen to focus on two core scientific disciplines, which are considered to have distinct approaches to aesthetics (Ivanova, 2017; McLeish, 2019; MacArthur, 2021). The survey was nationally representative of the target population in each country. More details on the study, including a methodological report, the data, and other study materials, are available in the public repository of this study (Vaidyanathan and Jacobi, 2022). As an incentive, a $20 e-gift card was given to survey respondents. Except for scientists in Italy, who had the option of taking the survey in Italian or English, most respondents answered the survey questions in English. This study received human subjects research approval from the Institutional Review Board of The Catholic University of America (21–0005). Informed consent was obtained from all participants.

### 3.2 Measures

#### 3.2.1 Dependent variables

**Aesthetic disposition**: The *dispositional positive emotions scale (DPES)* is a highly validated measure of the general disposition to experience the seven positive emotions of joy, contentment, pride, love, compassion, amusement, and awe (Shiota et al., 2007). The DPES is an established predictor of aesthetic emotions such as aesthetic intensity in the recent literature (Weigand and Jacobsen, 2022). For the current study, we focused on the awe dimension of the DPES as the conceptually relevant construct to the aesthetic components of the natural sciences (MacArthur, 2021). The six scales of the awe subdomain were: “I often feel awe,” “I see beauty all around me,” “I feel wonder almost every day,” “I often look for patterns in the objects around me,” “I have many opportunities to see the beauty of nature,” and “I seek out experiences that challenge my understanding of the world”; each of which were rated in response to the prompt “please indicate the extent to which you agree or disagree with the following statements in general.” Responses were scored on a Likert-type scale from 1 “disagree strongly” to 5 “agree strongly.” The mean score across response scales was then calculated. The Cronbach’s alpha for item reliability was acceptable at 0.76. Descriptive statistics of the three dependent variables are presented in Table 1.

**Table 1:** Descriptive statistics of the three dependent variables and their subitems.

	Mean	Percentage	Min	Max
Aesthetic disposition (DPES-awe) sub-scale items				
I often feel awe	3.3		1	5
I see beauty all around me	3.8		1	5
I feel wonder almost every day	3.2		1	5
I often look for patterns in the objects around me	3.9		1	5
I have many opportunities to see the beauty of nature	3.9		1	5
I seek out experiences that challenge my understanding of the world	3.9		1	5
Aesthetic experiences in scientific work scale items				
I felt pleased by encountering symmetry in scientific equations, models, or data	3.0		1	5
I felt pleased by the elegance of a scientific object (i.e., equation, model, experiment, etc.)	3.2		1	5
I felt surprised by discovering a hidden order or deeper systems underlying the phenomenon I was researching	2.9		1	5
I felt a sense of clarity as I saw how things fit together	3.3		1	5
Thinking about a scientific problem kept me awake at night	3.3		1	5
I felt my research opened up new mysteries for me to explore	3.3		1	5
I felt a sense of almost childlike delight or joy during my work	3.3		1	5
I felt grateful for learning something new	3.9		1	5
I felt my sense of self become somehow smaller in the face of what I was researching	2.4		1	5
I felt that I was in the presence of something grand	2.6		1	5
I felt a sense of reverence or respect about the things I was discovering	2.8		1	5
I was thrilled by a new insight	3.3		1	5
Aesthetic sensitivity in science scale items				
Writings of prominent scientists		36%	0	1
Scientific theories		61%	0	1
Phenomena that I study (e.g., cells, particles, etc.)		76%	0	1
The process of scientific research		52%	0	1
Teaching science		54%	0	1
Scientific journal articles		31%	0	1
Scientific conference presentations		30%	0	1

Survey-weighted descriptive statistics of scientists (biologists and physicists). Variables are later standardized in the regressions. Work and Wellbeing Study (2021). *N* = 3,092.

**Aesthetic experiences in scientific work:** The frequency of aesthetic experiences in scientific work scale is measured using the 12-item summative scale of frequency-anchored Likert items (0 = *never*, 1 = *rarely*, 2 = *a few times a year*, 3 = *a few times a month*, to 4 = *weekly or more*) capturing the domains of beauty (e.g., “I felt pleased by the elegance of a scientific object [i.e., equation, model, experiment, etc.]”), wonder (e.g., “Thinking about a scientific problem kept me awake at night”), and awe (e.g., “I felt that I was in the presence of something grand”) used by Jacobi et al. (2022). The items resemble and build upon approaches from other previously validated scales and studies (Shiota et al., 2006; Yaden et al., 2019). The Cronbach’s alpha was very good at 0.89.

**Aesthetic sensitivity in scientific work:** We conceptualized aesthetic sensitivity in science as the number of aspects of science in which scientists find beauty. Despite its exploratory nature, it seems to have face validity in measuring the sensitivity to most of the key aspects of work in science. Based on the survey question, “in which of the following aspects of work do you encounter *beauty* (however you define it)?,” scientists selected from a list of seven items. These were: “Writings of prominent scientists,” “scientific theories,” “phenomena that I study (e.g., cells, particles, etc.),” “the process of scientific research,” “teaching science,” “scientific journal articles,” “scientific conference presentations.” Selected items were scored as 1, and unselected items were scored as 0, resulting in a total range of the aesthetic sensitivity variable from 0 (none of the mentioned aspects of science are beautiful) to 7 (all mentioned aspects of science are beautiful). These aspects of science were derived from the philosophical literature on aesthetics in science (McLeish, 2019; MacArthur, 2021) and from qualitative interviews that preceded the study (Ecklund et al., 2019). Given the binary nature of the indicators for the aesthetic sensitivity in scientific work scale, item-response theory (IRT, see Supplementary material 1) analyses and principal component analyses (PCA), based on tetrachoric correlations (Supplementary material 2), were performed to evaluate the psychometric properties of this scale. The polychoric PCA of this scale was favorable with only one retained component (Eigenvalue of 3.92) whereas the other ones had Eigenvalues <1.

The analyses revealed that the item “my workplace,” which was asked alongside the other 7 included subitems, was not a good fit for the aesthetic sensitivity in science scale and was hence not included in the present scale.

#### 3.2.2 Independent variables

**Big Five personality types:** We employed the validated short BFI-10 scale (Rammstedt et al., 2013a,b) to measure the Big Five personality domains of extraversion, agreeableness, conscientiousness, neuroticism (emotional stability), and openness. Each domain consisted of two self-ratings in response to the leading question of “I see myself as someone who…”: The response items to this question were “reserved” and “outgoing/sociable” for extraversion, “generally trusting” and “tending to find faults with others” for agreeableness, “tending to be lazy” and “tending to do a thorough job” for conscientiousness, “relaxed/handling stress well” and “getting nervous easily” for neuroticism, and “having few artistic interests” and “having an active imagination” for openness. The responses were scored on a Likert-type response scale (1 “disagree strongly,” 2 “disagree somewhat,” 3 “neither agree nor disagree,” 4 “agree somewhat,” 5 “agree strongly”). The mean scores of the two responses for each of the five personality types were then computed. The correlations of the items and domains are shown in Supplementary material 3.

**Sociodemographic variables:** We included several sociodemographic control variables. These controls were gender (0 “men,” 1 “women”), country (1 “USA,” 2 “UK,” 3 “India,” 4 “Italy”), discipline (1 “physics,” 2 “biology,” 3 “other”), position/status (1 “postgraduate student,” 2 “postdoc” 3 “research scientist,” 4 “junior faculty,” 5 “mid-level faculty,” 6 “senior faculty”), age (continuous in years), survey wave (1 “May–June 2021,” 2 “August–October 2021”; capturing differences in exposure during COVID-19 pandemic between countries), the number of children under age 19 living in the household (continuous), and chronic health conditions (binary).

### 3.3 Analytic strategy

Aligned with the stated hypotheses, three major sets of regressions (Models 1–3) were run: Model 1 addressed the first hypothesis on associations between openness and neuroticism and higher, respectively lower, scores in aesthetic disposition. Models 2 addressed the second hypothesis on associations between openness and neuroticism with more frequent, respectively less frequent, aesthetic experiences in science. Models 3 the third hypothesis on associations between openness and neuroticism and higher, respectively lower, aesthetic sensitivity in science.

We employed multivariate ordinary least squares (OLS) regressions to model these relationships including the discussed covariates. The descriptive and regression statistics have been survey-weighted to represent the inferential population of scientists in the two disciplines and four countries. A similar study designs in terms of cross-cultural analyses of individual differences with the DPES is found in Nakayama et al. (2020) study.

To aid the interpretation and the comparability of results, all continuous variables (the three dependent variables, the personality predictor variables and age) were standardized. As only 350 of 3,442 respondents were dropped because they had a missing response on at least one of the relevant variables in this study, levels of missingness can be considered to be low for a survey of this kind during the pandemic.

## 4 Results

As presented in Table 2, the mean scores of the dependent variables before standardization were 3.66 on the DPES-awe sub-scale, 25.3 for the frequency of aesthetic experiences in scientific work, and 3.38 for aesthetic sensitivity in scientific work. The correlations between the three dependent variables (in the range of *r* = 0.27 to *r* = 0.39) were low to moderate, suggesting that the scales measure distinct aspects of the aesthetic.

In ranked order, scientists’ personality traits were highest on conscientiousness, openness, agreeableness, extraversion, and neuroticism. 54% of the weighted sample is found in the US, 27% in the UK, 10% in India, and 9% in Italy. Physicists comprised 52% of the sample, biologists constituted 38%, and other discipline 10%. Survey respondents tended to be relatively junior, with postgraduate students (29%) or postdocs (16%) representing over half the sample. 32% of the sample were women scientists, and 68% were men scientists. Around two-thirds of the sample experienced a significant stressor during the pandemic, and around half had seen their research projects put on hold.

**Table 2:** Descriptive statistics of the sample of scientists and variables in general.

	Mean	Percentage	Min.	Max.
Dependent variables				
Aesthetic disposition (DPES-awe)	3.7		1	5
Aesthetic experiences in scientific work	25.3		1	48
Aesthetic sensitivity in science	3.4			7
Focal independent variables				
Extraversion	2.9		1	5
Agreeableness	3.6		1	5
Conscientiousness	3.8		1	5
Neuroticism	2.9		1	5
Openness	3.7		1	5
Control variables				
Country				
USA		54%		
UK		27%		
India		10%		
Italy		9%		
Discipline				
Physics		52%		
Biology		38%		
Other (please specify)		10%		
Position/status				
Postgraduate student		29%		
Postdoc		16%		
Research scientist		5%		
Junior faculty		11%		
Mid-level faculty		12%		
Senior faculty		26%		
Gender: Women (ref. men)		32%		
Age	42.3		18	86
Survey wave: Wave 2 (ref. wave 1)		33%	1	2
Chronic health condition (ref. no such condition)		17%		

Survey-weighted descriptive statistics of scientists (biologists and physicists). Variables are later standardized in the regressions. Work and Wellbeing Study (2021). *N* = 3,092.

The regression results in Table 3 show the associations of the Big Five personality traits with dispositional and state aesthetic experiences in science while controlling for various sociodemographic and pandemic-related factors. All three hypotheses are fully confirmed: Openness is statistically significantly linked with higher scores on the standardized DPES-awe scale (*β* = 0.29 [0.24–0.35], *p* < 0.001), and more neuroticism is linked with lower scores (*β* = −0.12 [−0.19 to −0.05, *p* < 0.001]). Likewise, openness is associated with more frequent aesthetic experiences in scientific work (*β* = 0.13 [0.08–0.19], *p* < 0.001), while neuroticism is associated with less frequent aesthetic experiences in scientific work (*β* = −0.06 [−0.11 to −0.01, *p* < 0.05]). Similarly, openness is associated with higher aesthetic sensitivity in scientific work (*β* = 0.13 [0.08–0.17], *p* < 0.001), while neuroticism is associated with less frequent aesthetic experiences in science (*β* = −0.07 [−0.12 to −0.02, *p* < 0.01]).

**Table 3:** OLS regression results (Models 1–3) of aesthetic disposition, aesthetic experiences in scientific work, and aesthetic sensitivity in scientific work on personality traits among scientists.

	Aesthetic disposition (DPES-awe)	Aesthetic experiences in scientific work	Aesthetic sensitivity in scientific work
	Model 1	Model 2	Model 3
	OLS Beta coefficients	OLS Beta coefficients	OLS Beta coefficients
Big Five personality traits of scientists			
Extraversion	0.08+	−0.01	0.07*
	(−0.00 to 0.15)	(−0.09 to 0.08)	(0.00 to 0.13)
Agreeableness	0.11**	0.13***	0.15***
	(0.03 to 0.18)	(0.06 to 0.19)	(0.10 to 0.20)
Conscientiousness	0.13***	0.12***	0.05*
	(0.08 to 0.19)	(0.06 to 0.18)	(0.01 to 0.10)
Neuroticism	−0.12***	−0.06*	−0.07**
	(−0.19 to −0.05)	(−0.11 to −0.01)	(−0.12 to −0.02)
Openness	0.29***	0.13***	0.13***
	(0.24 to 0.35)	(0.08 to 0.19)	(0.08 to 0.17)
Control variables			
Gender: Women (ref. men)	−0.20**	−0.40***	−0.32***
	(−0.32 to −0.08)	(−0.56 to −0.24)	(−0.39 to −0.24)
Country: UK (ref. USA)	−0.23***	0.26**	−0.35***
	(−0.36 to −0.10)	(0.10 to 0.43)	(−0.44 to −0.27)
Country: India (ref. USA)	−0.30***	0.00	−0.13**
	(−0.43 to −0.18)	(−0.16 to 0.17)	(−0.22 to −0.04)
Country: Italy (ref. USA)	−0.04	0.02	−0.05
	(−0.14 to 0.07)	(−0.06 to 0.11)	(−0.19 to 0.08)
Discipline: Biology (ref. physics)	−0.16	0.05	−0.21*
	(−0.39 to 0.06)	(−0.26 to 0.37)	(−0.40 to −0.02)
Discipline: Other (ref. physics)	−0.30***	−0.19**	−0.05
	(−0.43 to −0.16)	(−0.31 to −0.07)	(−0.30 to 0.19)
Age (continuous)	−0.01	−0.42***	−0.49***
	(−0.20 to 0.18)	(−0.54 to −0.31)	(−0.63 to −0.36)
Position/status: Postdoc (ref. postgraduate student)	−0.28*	−0.51***	0.03
	(−0.50 to −0.06)	(−0.74 to −0.28)	(−0.22 to 0.27)
Position/status: Research Scientist (ref. postgraduate student)	−0.07	−0.40**	0.11
	(−0.27 to 0.12)	(−0.68 to −0.13)	(−0.12 to 0.33)
Position/status: Junior Faculty (ref. postgraduate student)	−0.01	−0.25*	−0.04
	(−0.24 to 0.22)	(−0.45 to −0.04)	(−0.29 to 0.22)
Position/status: Mid-level Faculty (ref. postgraduate student)	0.10*	−0.04	−0.17***
	(0.01 to 0.20)	(−0.16 to 0.08)	(−0.26 to −0.07)
Position/status: Senior Faculty (ref. postgraduate student)	−0.00	−0.01*	0.00
	(−0.01 to 0.01)	(−0.01 to −0.00)	(−0.01 to 0.01)
Survey wave: Wave 2 in August–October 2021 (ref. wave 1 in May–June 2021)	−0.01	−0.09*	−0.03
	(−0.10 to 0.08)	(−0.19 to −0.00)	(−0.14 to 0.08)
Chronic physical or mental health conditions (ref. no)	0.07	0.01	0.07
	(−0.08 to 0.21)	(−0.15 to 0.17)	(−0.06 to 0.21)
Observations	3,092	3,092	3,092
R-squared	0.21	0.15	0.14

Survey-weighted OLS regressions of scientists (biologists and physicists) and their aesthetic disposition (DPES-awe scale), frequency of aesthetic experiences in scientific work, and aesthetic sensitivity (index of aesthetic experiences across domains in scientific work). Standardized (β) regression coefficients for all dependent and continuous predictor variables. 95% confidence intervals in parentheses. ***p < 0.001, **p < 0.01, *p < 0.05, +*p* < 0.1. Work and Wellbeing Study 2021.

Unexpectedly large and consistently significant associations are found for agreeableness: Agreeableness has a very strong association with dispositional aesthetics (DPES-awe) (*β* = 0.11 [0.03–0.18], *p* < 0.01), an equally strong association with the frequency of aesthetic experiences in scientific work (*β* = 0.13 [0.06–0.19], *p* < 0.001) as openness, and the largest association of any of the personality traits with aesthetic sensitivity in scientific work (*β* = 0.15 [0.10–0.20], *p* < 0.001).

Conscientiousness also has a positive and significant association with DPES-awe and the frequency of aesthetic experiences in scientific work, but only a small one with aesthetic sensitivity in scientific work (*β* = 0.05 [0.01–0.10], *p* < 0.05). Extraversion is neither significantly linked with DPES-awe nor with the frequency of aesthetic experiences in model 2 (*β* = −0.01 [−0.09 to 0.08], *p* > 0.05), but significantly with aesthetic sensitivity (*β* = 0.07 [0.01–0.14], *p* < 0.05).

For sensitivity analyses, the sample was separately analyzed for women and men scientists, but the strong results for openness, neuroticism, and agreeableness were robust while conscientiousness sometimes no longer had a significant relationship at the *p* < 0.05 level with aesthetic sensitivity depending on the model specification. Each domain of the Big Five personality traits was regressed individually, but the results were largely similar. Given the timing of the survey during the COVID-19 pandemic, variables for having primary childcare responsibilities during the pandemic, COVID-19 impacts on research productivity, infection with the coronavirus, or other stressors during the pandemic were included as additional controls in each of the regressions, but the results of the regressions for the Big Five were not changed.

## 5 Discussion

The present study brings further clarity to the role of personality for aesthetic engagement given ambiguity in the literature, widens the frame of personality and aesthetics research beyond the traditional scope of the (largely visual) arts, and lays the groundwork for future examination of individual differences-specific implications for the way scientific research is itself studied, taught, and conducted. The findings of this study powerfully demonstrate that individual differences in scientists in terms of their personality traits are linked with dispositional and state aesthetic experiences in scientific work. Contrary to often-held stereotypes of science as being purely objective and detached, science here showed a dimension that is driven by aesthetic emotions and motivations. While previous literature mainly focused on arts-related populations such as museum visitors, this study provides insights into an entirely different population. In line with the literature experience (Rawlings et al., 2000; Keltner and Haidt, 2003; Thrash and Elliot, 2003; Shiota et al., 2007; Silvia et al., 2015; Atari et al., 2020), we find that the personality trait of openness is positively linked with dispositional aesthetic engagement as measured through the DPES-awe, and also positively associated with two state measures of the frequency of aesthetic experiences and aesthetic sensitivity in scientific work. Also, in line with the hypotheses, neuroticism negatively correlates with these three dependent variables of aesthetic engagement (Afhami and Mohammadi-Zarghan, 2018).

Importantly, we have identified the notable importance of agreeableness and conscientiousness among scientists with higher aesthetic dispositions and higher state aesthetic experiences in science. While there is concern about detrimental work conditions and a mental health crisis in science (Evans et al., 2018), and especially the lasting impacts of the COVID-19 pandemic on scientists (Gao et al., 2021), this study points to the promising benefits of agreeableness in particular. Scientists who score higher on agreeableness might be more collaborative, communicative, and supportive of their peers. It might be the case that agreeable scientists are more sensitive to the emotional and aesthetic aspects of scientific work. As such, agreeable scientists may be more likely to experience beauty, wonder, and awe when they discover a new phenomenon or observe a beautiful natural pattern. This emotional response may, in turn, enhance their motivation, creativity (Feist, 2010), and engagement in science. Agreeableness has been identified as a key wellbeing predictor (Anglim et al., 2020), and this positive relationship with wellbeing may be mirrored or even partly operate through aesthetic engagement.

## 6 Conclusion, limitations, and implications

### 6.1 Main findings and contributions

Our aesthetic engagement and personality framework offers an integrative model to explain how personality traits influence perception and engagement with aesthetics across different life contexts. Focusing on a non-arts population which is stereotypically seen as purely rational and dry, namely scientists in the fields of biology and physics in the US, UK, India and Italy, the statistical results of this study confirm this theoretical framework and show that scientists’ personality traits of openness, agreeableness, and conscientiousness are strongly and positively linked with higher trait aesthetic disposition (as indicated by the DPES-awe) as well as higher state aesthetic engagement in terms of the frequency of aesthetic experiences in scientific work and in terms of aesthetic sensitivity in science (the contexts in which beauty, wonder, and awe are experienced in science). Whereas extraversion was only weakly associated with the three indicators of trait and state aesthetic engagement, the relationships with neuroticism were strongly negative as predicted by the literature.

The study’s strengths are the novel population of scientists in the natural sciences under which individual differences and personality factors are studied in relation to dispositional aesthetics, aesthetic experiences, and aesthetic sensitivities. The study adds to the slim corpus of empirical studies that have examined all five personality factors with a general conceptualization of aesthetic beauty, wonder, awe (frequency of aesthetic experiences), and aesthetic sensitivity in a non-arts-related population. This is the largest-ever study of beauty and aesthetics in science, enabling novel analyses of this topic with the highest quality data and instruments. The four countries provide a global perspective and account for some cultural differences in aesthetic engagement. The statistical models have employed complex survey weighting and extensive control variables.

### 6.2 Limitations

The study is limited by its cross-sectional design, implying that the findings cannot be directly interpreted as causal and ought to be replicated with different data. While the academic literature generally suggests that personality traits are relatively stable in adulthood and, therefore, temporally antecedent to aesthetic experiences in Cobb-Clark and Schurer’s (2012) work, there is also evidence that they can change over time (Debast et al., 2014), even differentially across traits. The scale for the frequency of aesthetic experiences at work is somewhat crude as those scientific work experiences may also be shaped by other forces such as job pressures, poor mentorship, workplace mistreatment, institutional policies, or other outside factors. A more experimental research design may be different fruitful where specific aesthetic emotions are manipulated and measured in real-time (Williams et al., 2022). While the Big Five scale has been well-validated (Rammstedt et al., 2013a), it is very short, and valuable nuances may be discovered on a larger scale that would allow for different analyses, such as structural equation modeling.

### 6.3 Implications and future research

Despite these limitations, there are multifaceted implications of the finding that the Big Five personality traits relate to aesthetic engagement in science: The personality of scientists could affect their motivation, their wellbeing (Jacobi et al., 2022), their collaboration, and even scientific communication (Lewis and Wai, 2021). As such, scientists with personalities who are more likely to experience emotions like beauty, wonder, and awe may be more motivated to engage in research, communicate their findings, and pursue new and creative ideas. Collaborative research and communication may be improved by scientists who appreciate each other’s research and its aesthetic aspects. The aesthetic engagement and personality framework can guide future research in this area and offer a theoretical foundation for interventions aimed at enhancing wellbeing, especially in workplace contexts, such as creating environments that enhance the interaction between personality and aesthetic engagement.

Furthermore, future research could employ the two original scales (frequency of aesthetic experiences in scientific work and aesthetic sensitivity in science scales) that were developed with this data set, replicate these scales and findings with other data, or test the stated relationships in other populations and settings. Moderation effects between the Big Five personality traits as well as more nuances between the three positively correlated personality traits of openness, agreeableness, and conscientiousness with aesthetic engagement could be explored.

## Data availability statement

The datasets presented in this study can be found in online repositories. The names of the repository/repositories and accession number(s) can be found at: https://osf.io/jp86u/.

## Ethics statement

This study received human subjects research approval from the Institutional Review Board of the Catholic University of America (21–0005). Informed consent was obtained from all participants. The studies were conducted in accordance with the local legislation and institutional requirements. The participants provided their written informed consent to participate in this study.

## Author contributions

CJ planned the study, identified suitable variables, conducted the statistical analyses, and wrote the manuscript. PV helped write the literature review and edited the manuscript. ZJ conducted the literature review and advised on the manuscript. BV envisioned the study in general, provided the dataset and advised on the manuscript. All authors contributed to the article and approved the submitted version.
