# Supplementary material for: Individual differences in scientists’ aesthetic disposition, aesthetic experiences, and aesthetic sensitivity in scientific work
Source: Front Psychol. 2024 Jan 8;14:1197870. doi: 10.3389/fpsyg.2023.1197870 (PMC10800433; doi:10.3389/fpsyg.2023.1197870)

IRT-analyses (two-parameter logistic model) of the aesthetic sensitivity in science scale.
The theta scores of the seven binary indicators of the aesthetic sensitivity in science appear to be favorable. The item beautywork_7 (“scientific journal articles”) appears to be the one with the highest difficulty value.

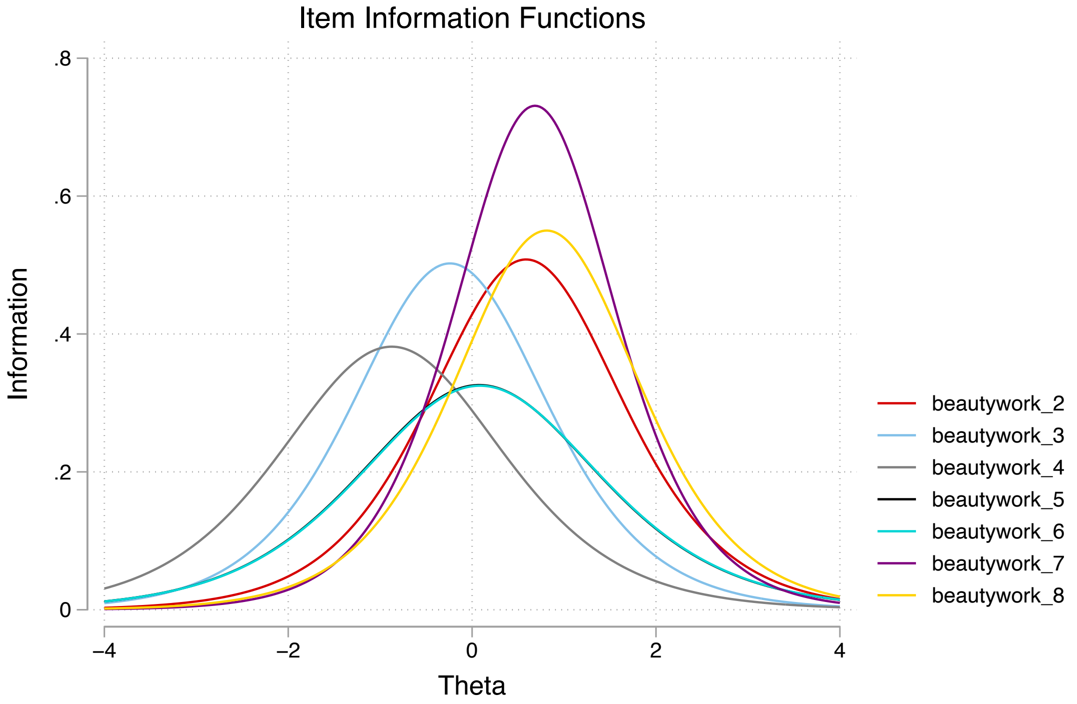

IRT-analyses of the aesthetic sensitivity in science scale.
The first item (“beautywork_1”, i.e., in the workplace) did not fit well the other items was dropped.

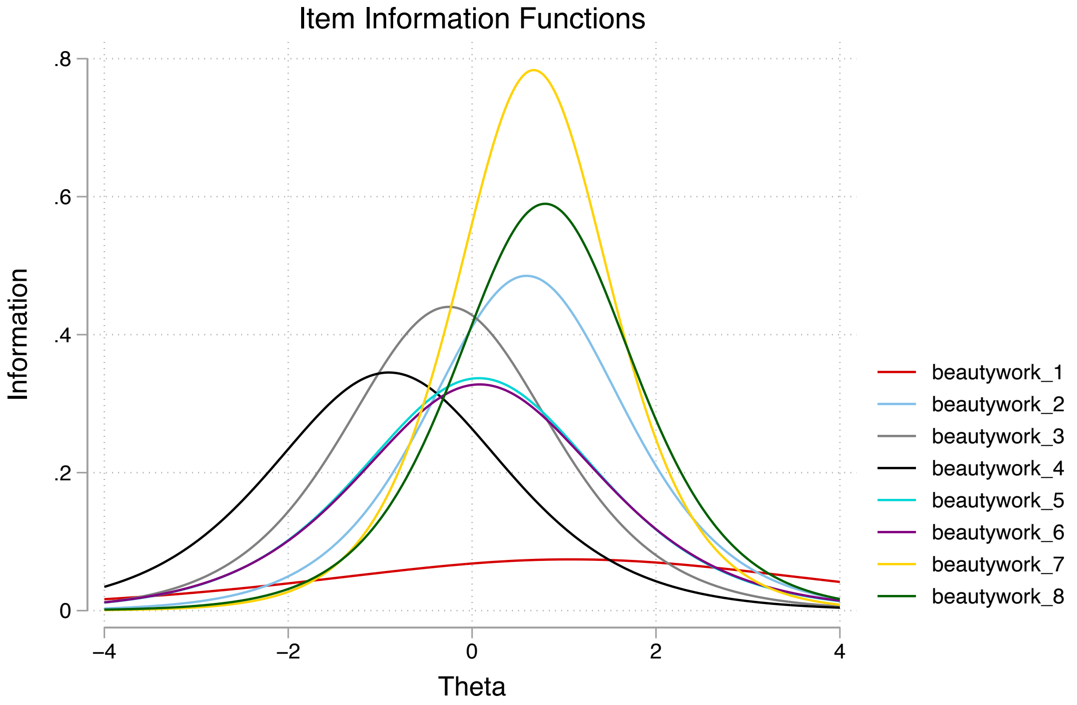

Supplement: Supplementary file 1 [file Data_Sheet_1.PDF]
