# Supplementary material for: Individual differences in scientists’ aesthetic disposition, aesthetic experiences, and aesthetic sensitivity in scientific work
Source: Front Psychol. 2024 Jan 8;14:1197870. doi: 10.3389/fpsyg.2023.1197870 (PMC10800433; doi:10.3389/fpsyg.2023.1197870)
Supplement: Supplementary file 2 [file Data_Sheet_2.PDF]

Polychoric correlation coefficients of the subitems of the aesthetic sensitiy in science scale

		(1)	(2)	(3)	(4)	(5)	(6)	(7)
(1)	Writings of prominent scientists	1						
(2)	Scientific theories	0.51*	1					
(3)	Phenomena that I study (e.g., cells, particles, etc.)	0.36*	0.49*	1				
(4)	The process of scientific research	0.31*	0.37*	0.32*	1			
(5)	Teaching science	0.31*	0.36*	0.34*	0.40*	1		
(6)	Scientific journal articles	0.49*	0.34*	0.29*	0.39*	0.35*	1	
(7)	Scientific conference presentations	0.37*	0.29*	0.34*	0.33*	0.38*	0.62*	1

Note: Work and Well-Being Study (2021). N = 3,092.
