# Supplementary material for: Individual differences in scientists’ aesthetic disposition, aesthetic experiences, and aesthetic sensitivity in scientific work
Source: Front Psychol. 2024 Jan 8;14:1197870. doi: 10.3389/fpsyg.2023.1197870 (PMC10800433; doi:10.3389/fpsyg.2023.1197870)
Supplement: Supplementary file 3 [file Data_Sheet_3.PDF]

Pairwise correlation of the Big Five subitems

	I see myself as someone who is…	(1)	(2)	(3)	(4)	(5)	(6)	(7)	(8)	(9)	(10)
(1)	is reserved	1									
(2)	is generally trusting	-0.022	1								
(3)	tends to be lazy	0.098***	0.038**	1							
(4)	is relaxed, handles stress well	-0.066***	0.136***	-0.061***	1						
(5)	has very few artistic interests	-0.005	0.025	0.119***	0.105***	1					
(6)	is outgoing, sociable	-0.450***	0.202***	-0.097***	0.208***	-0.003	1				
(7)	tends to find fault with others	0.077***	-0.205***	0.146***	-0.101***	0.049***	-0.081***	1			
(8)	does a thorough job	0.093***	0.156***	-0.295***	0.085***	-0.065***	0.087***	-0.025	1		
(9)	gets nervous easily	0.198***	-0.023	0.209***	-0.409***	0.035*	-0.153***	0.217***	-0.022	1	
(10)	has an active imagination	-0.030*	0.161***	-0.071***	0.122***	-0.178***	0.165***	-0.050***	0.190***	0.01	1

Note: Work and Well-Being Study (2021). N = 3,092.
